# Reversing the Tumor Target: Establishment of a Tumor Trap

**DOI:** 10.3389/fphar.2019.00887

**Published:** 2019-08-12

**Authors:** Mathie Najberg, Muhammad Haji Mansor, Frank Boury, Carmen Alvarez-Lorenzo, Emmanuel Garcion

**Affiliations:** ^1^CRCINA, INSERM, Université de Nantes, Université d’Angers, Angers, France; ^2^Departamento de Farmacología, Farmacia y Tecnología Farmacéutica, R + D Pharma Group (GI-1645), Facultad de Farmacia, Universidade de Santiago de Compostela, Santiago de Compostela, Spain; ^3^Center for Education and Research on Macromolecules (CERM), Université de Liège, Liège, Belgium

**Keywords:** tumor cell migration, tumor trap, biomimetic trap, cancer therapy, premetastatic niche recruitment

## Abstract

Despite the tremendous progress made in the field of cancer therapy in recent years, certain solid tumors still cannot be successfully treated. Alongside classical treatments in the form of chemotherapy and/or radiotherapy, targeted treatments such as immunotherapy that cause fewer side effects emerge as new options in the clinics. However, these alternative treatments may not be useful for treating all types of cancers, especially for killing infiltrative and circulating tumor cells (CTCs). Recent advances pursue the trapping of these cancer cells within a confined area to facilitate their removal for therapeutic and diagnostic purposes. A good understanding of the mechanisms behind tumor cell migration may drive the design of traps that mimic natural tumor niches and guide the movement of the cancer cells. To bring this trapping idea into reality, strong efforts are being made to create structured materials that imitate myelinated fibers, blood vessels, or pre-metastatic niches and incorporate chemical cues such as chemoattractants or adhesive proteins. In this review, the different strategies used (or could be used) to trap tumor cells are described, and relevant examples of their performance are analyzed.

## Introduction

**Figure 1:**
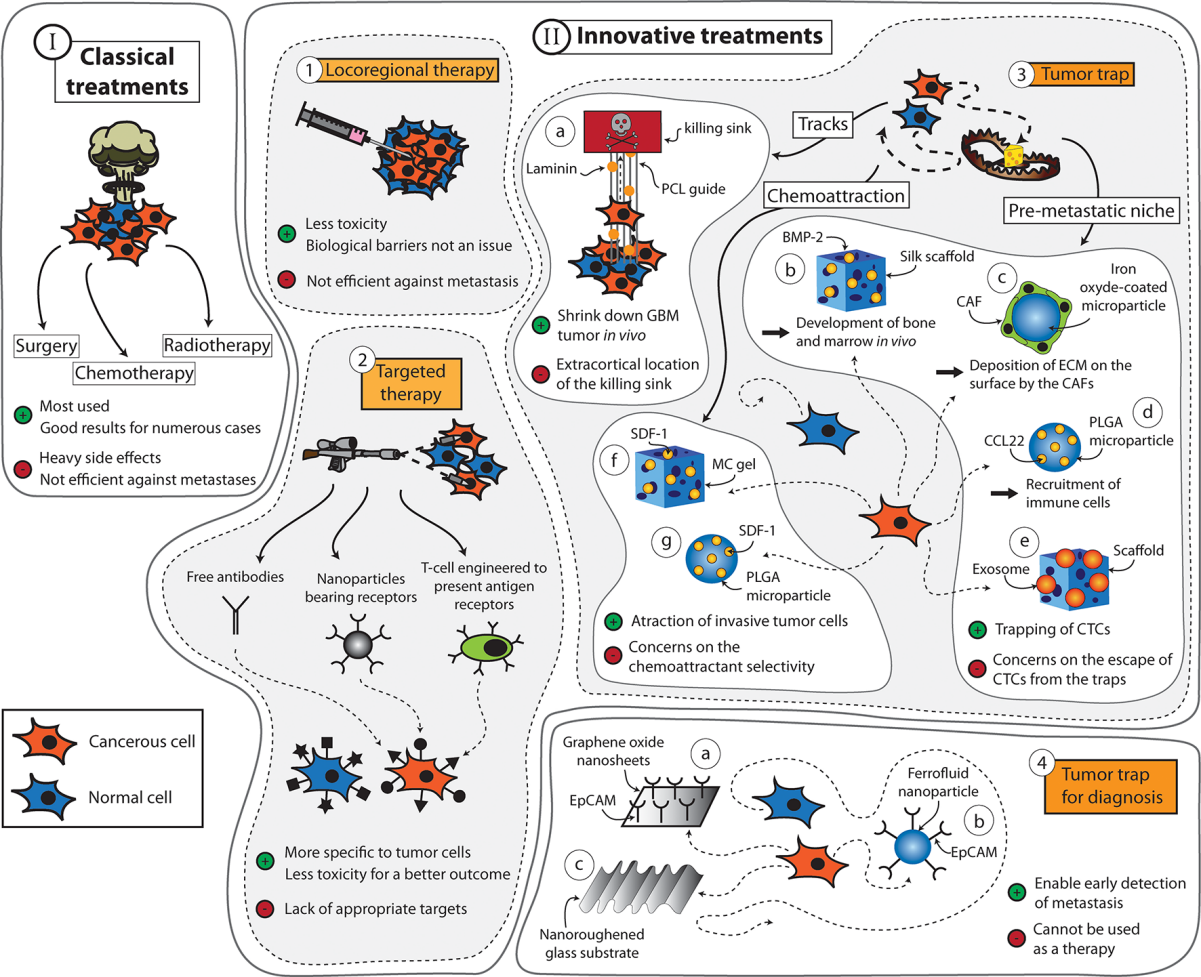
Summary of the strategies that can be applied to fight cancers. (Scheme I) The classic treatments used for cancers are surgery, chemotherapy, and radiotherapy. (Scheme II) Innovative treatments include (1) locoregional therapy, (2) targeted therapy, and (3) tumor traps, among others. Tumor traps can be designed to take advantage of the migration pathways used by the tumor cells. It includes the use of tracks [(a) system developed by Jain et al. (2014) using aligned PCL fibers coated with laminin]. Tumor traps can be designed as synthetic pre-metastatic niches [(b) system developed by Seib et al. (2015) using a silk scaffold loaded with bone morphogenic protein 2 (BMP-2) capable of developing bone and marrow *in vivo*, (c) system developed by De Vlieghere et al. (2015) using iron oxide-coated microparticles encapsulating cancer-associative fibroblasts (CAFs) that continuously deposit ECM on the surface, (d) system developed by Azarin et al. (2015) using poly(lactide-co-glycolic acid) (PLGA) through the induction of the immune system by the CCL22 chemokine, and (e) system developed by De La Fuente et al. (2015) using a three-dimensional scaffold loaded with exosomes]. Finally, tumor traps can use chemoattractive molecules [(f) system developed by Giarra et al. (2018) using a methylcellulose (MC) thermo-responsive hydrogel loaded with stromal derived factor-1 (SDF-1) and (g) the system developed by Haji Mansor et al. (2018) using SDF-1 encapsulated in PLGA nanoparticles]. (4) Tumor traps can also be used for the early detection of metastasis [(a) system developed by Yoon et al. (2013) using graphene oxide nanosheets, (b) CELLSEARCH^®^ CTC test (CELLSEARCH Circulating Tumor Cell, 2019) is a device using ferrofluid nanoparticles with EpCAM antibodies, and (c) system developed by Chen et al. (2016) using a nanoroughened glass substrate].

For many decades, surgery, radiotherapy, and chemotherapy have served as the mainstay trident in the fight against cancer (**Figure 1** Scheme I). During this period, the prognosis of many types of cancer has been significantly improved (Brustugun et al., 2018; Zeng et al., 2018; Iacobucci, 2019; Trama et al., 2019). However, the widespread use of these treatments has also uncovered several major limitations. For example, the feasibility of surgery is very much dependent on the localization and the size of the tumor. The procedure is also contraindicated in patients with poor clinical performance. As for radiotherapy and chemotherapy, these treatments are often implicated with serious side effects that, in some cases, may outweigh their potential therapeutic benefits. Moreover, these treatments lack the capacity to prevent metastases, which are responsible for roughly 90% of cancer-associated deaths (Rankin and Giaccia, 2016).

Numerous studies in the quest of improving cancer treatments are driven by the concept of “magic bullet’’ (**Figure 1** Scheme II-2) put forward by the German scientist Paul Ehrlich (Strebhardt and Ullrich, 2008). If radio- and chemotherapy are considered as weapons of mass destruction, Ehrlich’s strategy can be regarded as the sniper of cancer therapy. This concept is mainly based on the idea of increasing the bioavailability and specificity of vector-associated active agents in the body while limiting their premature degradation and toxicity. In the context of anticancer approaches, the success of selective therapies depends on the discovery of targeting elements that, when coupled with active ingredients and/or diagnostic cues, enable the recognition of well-characterized molecules, cells, or tissues. For example, Adcetris^®^ targets the antigen CD30 in the treatment of Hodgkin’s lymphoma, and Kadcyla^®^ targets HER2, which is present in about 20% of breast cancer patients (Kim and Kim, 2015). Nevertheless, the discovery of appropriate targets that are specific to tumor cells remains a challenging task, despite the significant advancements made in the field of genomics and proteomics in recent decades.

Fortunately, a plethora of new therapies are being approved regularly for the treatment of cancer. Among them is the use of locoregional therapies (**Figure 1** Scheme II-1) that includes Nanotherm^®^ (MagForce) that involves injection of magnetic nanoparticles inside the tumor or into the resection cavity. A magnetic field is then applied to generate heat *via* the nanoparticles and kill the cancerous cells locally (Maier-Hauff et al., 2011). It is currently licensed in Europe for the treatment of brain tumors and has received FDA approval in February 2018 to be used in clinical trials involving prostate cancer patients (MagForce, 2018). Another example is Optune^®^ (Novocure Ltd), a tumor-treating field (TTF) device composed of electrodes that can be placed on the patients’ scalp and connected to a generator to deliver a low-intensity electric field of 200 kHz (Taphoorn et al., 2018). It is believed to exert anticancer effects by disrupting the division of tumor cells (Giladi et al., 2015). The device has been approved for the treatment of glioblastoma and shown to increase the median survival from 15 to 21 months when used on top of the standard treatments for this cancer (Stupp et al., 2017). However, many countries and insurance companies do not cover the cost of this treatment, and the clinical adoption of this technology remains limited due to concerns regarding the lack of understanding of the device’s exact mechanism of action. Moreover, some skepticism exists toward the legitimacy of the device approval process due to the poor consideration of any placebo effect during the clinical trial phase (Fabian et al., 2019).

Among the numerous classes of novel anticancer treatments entering the market, cancer immunotherapy is arguably the one that is currently attracting the highest level of attention (**Figure 1** Scheme II-2). This class of treatment aims to treat cancer through artificial stimulation of the patient’s immune system (Zhang and Chen, 2018). The most cutting-edge subset of this type of treatment is the chimeric antigen receptor (CAR) T-cell immunotherapy, which involves harvesting T cells from a patient and genetically modifying these cells to express a receptor that can bind to a tumor antigen before injecting them back into the patient (Feins et al., 2019). CAR-T cell immunotherapy made its debut in the clinic in August 2017 when Kymriah^®^ (Novartis) was approved by the FDA for the treatment of B-cell acute lymphoblastic leukemia (BCALL) (FDA, 2017a). This was followed by the approval of Yescarta^®^ (Gilead Sciences) in October of the same year for the treatment of diffuse large B-cell lymphoma (FDA, 2017b). Both Kymriah^®^ and Yescarta^®^ exert their effects by targeting CD19 antigen (Zheng et al., 2018). However, there are numerous ongoing clinical studies that explore the feasibility of targeting other antigens including PD-L1 (ClinicalTrials.gov Identifier NCT03672305, NCT03198052, NCT03330834), EpCAM (ClinicalTrials.gov Identifier NCT03013712, NCT03563326, NCT02729493), and CD123 (ClinicalTrials.gov Identifier NCT03796390, NCT02937103, NCT03672851). Many of these trials also attempt to evaluate the efficacy of CAR-T cell immunotherapy against solid tumors to expand its indication beyond certain blood cancers. More comprehensive reviews on the current status and future directions of CAR T-cell immunotherapy as well as other subsets of cancer immunotherapy such as immune checkpoint inhibitors and cancer vaccines can be found elsewhere (Dougan et al., 2018; Li et al., 2018; Sambi et al., 2019).

Despite the continuous increase in the number of novel anticancer treatments entering the clinic, local recurrence in previously healthy tissues seen in many cases of solid tumors remains an unsolved conundrum among clinicians and researchers alike. Development of new therapies for *in situ* control of the disease, while avoiding the problems of biological barriers and systemic toxicity, still proves to be a formidable task. Thus, in parallel to the innovative approaches mentioned previously, the idea of trapping infiltrative or circulating tumor cells (CTCs) within a confined area to facilitate their removal for therapeutic or diagnostic purposes has risen (**Figure 1** Scheme II-3,4). Over the last years, this concept has developed progressively. The aim is twofold: a) to avoid the uncontrolled dissemination of tumor cells and b) to efficiently prevent the phenomenon of epithelial–mesenchymal transition (EMT) or development of metastases. The concept is largely inspired by the “ecological trap” theory (van der Sanden et al., 2013). By considering cancers as ecosystems, it is possible to develop tumor traps not only for the infiltrative tumor cells, but also for the CTCs that are responsible for metastasis. However, imitating the traditional features of a natural habitat or niche for tumor cells and directing their migration pathways present numerous physical and biological challenges. The focus of this review will be on understanding the mechanisms of tumor cell migration and how this knowledge can be used to capture them, keeping in mind that different tumors are likely to utilize different mechanisms.

## Migration of Tumor Cells

**Figure 2:**
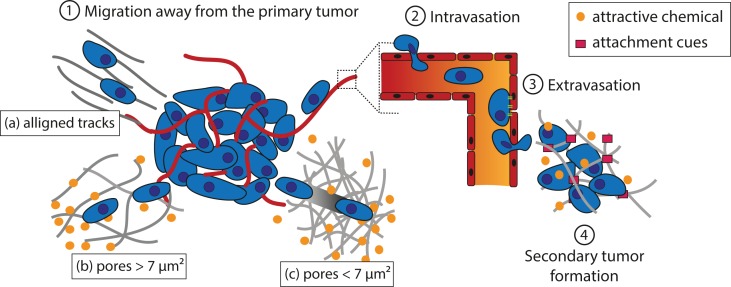
Schematic representation of theorized paths of cancer cell migration away from the primary tumor. (1) Migration away from the primary tumor. Tumor cells can follow aligned tracks (a), or gradients of chemoattractant in solution (chemotaxis) or fixed on a substrate (haptotaxis) through the ECM. If the cross-sectional area of the interfibrillar pore is more than 7 µm², degradation of the matrix is not needed (b); otherwise, a leader cell creates a path for the following cells thanks to MMPs (c). (2) Tumor cells adhere to a vessel and intravasate to reach the blood or lymphatic stream in which they circulate. (3) Once they reach a location where they can adhere to the vessel wall, the cells extravasate. (4) If the environment is favorable, a secondary tumor grows.

Tumor cells must cover a great distance on their journey to form metastases (**Figure 2**). The first step of the process is to migrate away from the primary tumor. Tumor cells can follow aligned tracks a), or gradients of chemoattractant in solution (chemotaxis) or fixed on a substrate (haptotaxis) through the extracellular matrix (ECM) b). If the cross-sectional area of interfibrillar pores is more than 7 µm², degradation of the matrix is not needed for cell movement. Alternatively, a leader cell can open a path for the following cells by virtue of the matrix metalloproteinase (MMP) activity c). The second step is to intravasate into the bloodstream or the lymphatic system in which the tumor cells will transit through the circulation. Third, cells extravasate to secondary tissues once they reach a location where they can adhere to the walls of the vessel. The fourth and final step deals with the formation of a secondary tumor. This only occurs if the environment is favorable to tumor growth.

The different strategies implemented to mislead these cells into a trap are described in next sections. These strategies exploit the current knowledge on cancer cell migration and metastasis and the specificities of each type of tumor.

### Migration Away From the Primary Tumor

The physical interactions between the ECM and cancer cells play a key role in allowing the cells to start migrating. Cancer cells may undergo an EMT to acquire a motile phenotype (Polyak and Weinberg, 2009). This translates into the loss of intracellular adhesion molecules such as E-cadherin and cytokeratins, resulting in detachment of the cells from the primary tumor, and an overexpression of MMP on their surface that allows the cells to digest laminin and collagen IV to progress in the dense ECM (Polyak and Weinberg, 2009). These changes are thought to be related to the stiffness of the matrix around the tumor, which is of higher values than that of normal tissues (Wozniak et al., 2003; Paszek et al., 2005; Kumar and Weaver, 2009; Levental et al., 2009). For example, the stiffness of GBM tissues is of ∼25 kPa. while normal brain tissues have a stiffness of 0.1 to 1 kPa (Wang et al., 2014). Wang et al. investigated the effect of matrix stiffness on GBM cells and found that an increase in matrix rigidity could induce an upregulation of MMP-1, Hras, RhoA, and ROCK1 (Wang et al., 2014), which are involved in increasing cell motility (Pulukuri and Rao, 2008; Liu et al., 2014; Wu et al., 2016; Li et al., 2017). Another physical factor that governs the dissemination of cancer cells is the architecture of the extracellular environment, which includes pores of a diameter ranging from less than 1 to 20 µm (Wolf et al., 2009). Matrix degradation is usually required for cancer cell migration to occur when the cross-sectional area of the interfibrillar pore is less than 7 µm², which corresponds to about 10% of the nuclear cross-section of cancer cells (Wolf et al., 2013). Above this value, cells can undergo deformation to migrate through the ECM.

Apart from the porosity of the ECM, the spatial arrangement of the matrix fibers near the primary tumor sites can also influence the motility of tumor cells; aligned fibers offer tracks that are more conducive to migration (Provenzano et al., 2008; Paul et al., 2017). These tracks are found along the ECM fibers in the interstitial space, between the muscle and nerve fibers, and along or within the vasculature of organs, among others (Gritsenko et al., 2012). Moreover, it has been observed that leader tumor cells are able to align collagen fibers to assist the migration of the following cells (Provenzano et al., 2006). In addition to creating the required physical space, these tracks also facilitate cancer cell migration by providing relevant molecular guidance. For example, cancer cells can be guided toward laminin and hyaluronan molecules in the ECM by their integrins and CD44 receptors, respectively, and also *via* haptotaxis by chemokines and growth factors immobilized along the tracks (Aznavoorian et al., 1990; Gritsenko et al., 2012). Jain et al. took inspirations from these biological phenomena and designed a scaffold to guide GBM cells toward a killing sink in an extracortical location (**Figure 1** Scheme II-3-a) (Jain et al., 2014). They utilized aligned poly-L-lysine and laminin-coated polycaprolactone (PCL) nanofibers (10 μm thick) encased in a PCL/polyurethane support (2.4 mm diameter) to imitate the white matter tracts (Bernstein and Woodard, 1995; Giese et al., 2003). The killing sink was composed of a collagen-based hydrogel conjugated to the chemotherapeutic agent cyclopamine. With this approach, the tumor mass of induced GBM in mice could be reduced. However, despite the positive results, this strategy as itself has limited clinical appeals, as the establishment of an extracortical sink in human patients may invite numerous technical difficulties. Instead, exploiting the local (intracortical) migration of the cancer cells may be a more translatable strategy to develop an efficient tumor trap for this cancer.

### Intravasation and Tumor Cell Circulation

Tumor cells can circulate through the blood and lymphatic vessels on their journey to form a secondary tumor distant from the primary site (Chiang et al., 2016). This requires the cells to intravasate by passing through the endothelial cell junctions. Intravasation into the blood vessels occurs frequently due to the leaky nature of tumor vasculature. In addition, it has been observed *in vivo* that metastatic cells are able to polarize toward blood vessels. A possible explanation to this phenomenon is that these cells have an increased expression of epidermal growth factor (EGF) and/or colony-stimulating factor 1 (CSF-1) receptors. Thus, they migrate toward a gradient of EGF or CSF-1 released by the macrophages lining the blood vessels (Wyckoff et al., 2000; Wyckoff et al., 2004). However, it is still easier for tumor cells to enter the lymphatic system, as the surrounding ECM network is easier to penetrate and that the endothelial junctions are looser (Wong and Hynes, 2006). Either route can lead to blood vessel dissemination since the lymphatic circulation drains into the blood. As the lymphatic fluid is filtered by the lymph nodes, tumor cells are invariably invading them, starting with the nearest (Nathanson, 2003).

**Figure 3:**
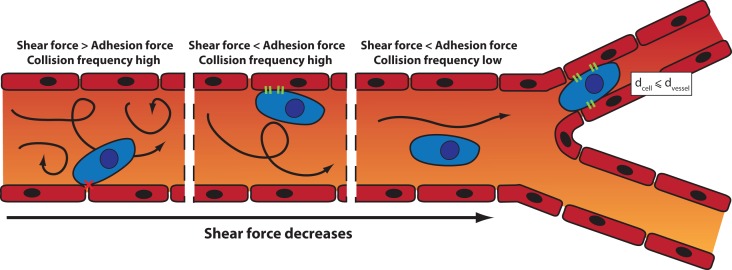
Mechanisms of arrest of a CTC: the influence of the shear force and the blood vessel diameter on the site of CTC extravasation.

Once in the blood circulation, the trajectory of the tumor cells is influenced by the blood flow, the diameter of the blood vessels, and the intercellular adhesion (Wirtz et al., 2011). Two mechanisms can lead to the arrest of a CTC: physical occlusion and cell adhesion (**Figure 3**). Physical occlusion occurs when the diameter of the blood vessel is smaller or equal to the one of the CTC (usually around 10 µm). This has been observed in the brain by real-time imaging in a mouse model (Kienast et al., 2010). Adhesion of CTCs to the vessel walls occurs when there is a balance between the adhesion force and the shear force exerted inside the blood vessel (Zhu et al., 2008). When the shear force increases, the collisions between cells and the vessel wall increase, which in turn enhances the likelihood of cell adhesion. However, if the shear force is too high, turbulences may prevent the adhesion.

It is therefore possible to capture CTCs by designing a device with strong adhesive cues. Yoon et al. (2013) designed a microfluidic device consisting of graphene oxide nanosheets fixed onto a patterned gold surface to capture CTCs in early-stage cancer for analytical purposes (**Figure 1** Scheme II-4-a). The nanosheets were functionalized with epithelial cell adhesion molecule (EpCAM) antibody to capture CTCs. Blood samples were retrieved from healthy donors and mixed with labeled human breast cancer cell lines MCF-7 and Hs-578T and the human prostate cancer cell line PC-3. This microfluidic device captured more than 70% of the cancer cells in the prepared blood sample with high specificity. A similar principle was also implemented in the design of CELLSEARCH^®^ CTC Test, the first and only clinically validated and FDA-approved blood test for enumerating CTCs (CELLSEARCH Circulating Tumor Cell, 2019). It allows for early assessment of patient prognosis as well as follow-up of the patient. The test constitutes the use of ferrofluid nanoparticles with EpCAM antibodies that bind to CTCs (**Figure 1** Scheme II-4-b). Once magnetically separated from the rest of the blood sample, cells are stained to discriminate CTCs from leukocytes that can copresent in the sample. Working within the same domain of research, Chen et al. (2016) designed a nanoroughened glass substrate to capture CTCs based on their stronger adhesion capacity compared with normal blood cells (**Figure 1** Scheme II-4-c). Such a working principle makes this device useful for capturing CTCs regardless of their surface marker expression profile, which is known to vary according to the type of cancer, patient demographics, and the state of the disease. It is indeed well discussed in the literature that the EMT process may lead to reduction in the epithelial cell adhesion molecule (EpCAM) expression in CTC (Hyun et al., 2016). The capture of CTC using EpCAM antibody alone may lead to an underestimation of the CTC number in the blood. With this device, more than 80% of cancer cells in whole blood samples from mice with induced breast cancer or lung cancer were captured independently of their EpCAM expression. Based on these findings, it is evident that a number of approaches can be adopted to capture CTCs to enable early detection of metastasis, although most of them are still far from translation to the clinic.

### Extravasation and Secondary Tumor Formation

At the end of their time in blood circulation, the CTCs that survived and adhered to the blood vessel walls extravasate, and a fraction of these proceeds to form a secondary tumor. It has been shown experimentally that only about 0.01% of the cells in the circulation system eventually contribute to metastatic colonization (Fidler, 1970). The location at which they stop and grow into a secondary tumor is not believed to be randomly determined, and the reasons driving the selection of a particular site are still being investigated. In 1889, Paget et al. hypothesized that metastasizing cancer cells are like seeds that can only grow in the proper soil (Paget, 1889). Indeed, it has been observed that invasive cancer cells tend to migrate toward certain preferred sites of metastasis, a phenomenon that has been coined as “tissue tropism” (Seib et al., 2015). More recent studies revealed that the formation of certain microenvironments termed as pre-metastatic niches is crucial to the subsequent formation of metastatic tumors. These microenvironments consist of inflammatory immune cells, stromal cells, ECM proteins, tumor-secreted exosomes, and homing factors (Aguado et al., 2017). Tumor-secreted exosomes are sent to prime the niche at a target organ (often lungs, liver, brain, bone, and lymph nodes) by attracting bone marrow-derived cells (BMDCs) as well as increasing the proliferation of fibroblast-like stromal cells (Peinado et al., 2012). BMDCs include CD11b^+^ myeloid cells, myeloid-derived suppressor cells, neutrophils, tumor-associated macrophages, and regulatory T cells. They are known to create an attractive site for metastasizing cells, and the presence of VEGFR1-positive BMDCs can serve as a predictor for the arrival of tumor cells (Kaplan et al., 2005). Moreover, the establishment of a pre-metastatic niche is associated with an increased secretion of inflammatory cytokines and chemokines (Hiratsuka et al., 2006; Darash-Yahana et al., 2009; Brennecke et al., 2014). The increasing understanding of the pre-metastatic niches and their roles in welcoming metastatic dissemination has inspired scientists to create synthetic niches as a means to trap migrating cancer cells.

#### Creation of a Synthetic Pre-metastatic Niche to Trap CTCs

Many different strategies have been explored to engineer pre-metastatic niches. For example, Seib et al. (2015) developed a tumor trap for the metastasizing cells of breast and prostate cancers by imitating the red bone marrow microenvironment (**Figure 1** Scheme II-3-b). The strategy was adopted based on the knowledge that the bone was the preferred site of colonization in more than 60% of cases of metastasis for primary breast cancer and 73% for primary prostate cancer (Weiss, 1992). Evidence shows that red bone marrow attracts migrating cancer cells *via* chemotaxis with stromal derived factor-1 (SDF-1) (Taichman et al., 2002) and CXCL16 (Lee et al., 2013). It also provides adhesion sites that interact with tumor cell surface molecules such as annexin2 (Shiozawa et al., 2008), growth arrest-specific 6 (GASP-6) (Shiozawa et al., 2010), CD44 (Hill et al., 2006), integrins (such as VLA-4, VLA-5, and LFA-1), and cadherins. Moreover, the bone marrow microenvironment is composed of osteoblasts, osteoclasts, stromal cells, stem cells, and mineralized bone marrow surrounded by a rich vascular bed, making it a perfect site for tumor growth (Mishra et al., 2011). To imitate the red bone marrow, Seib et al. designed a silk fibroin scaffold loaded with bone morphogenetic protein 2 (BMP-2) that is capable of developing bone and marrow *in vivo*. After implantation into the mammary fat pads of mice with induced breast or prostate tumor, no effect on the primary tumor growth was observed. However, metastatic growth could be seen taking place in the functionalized scaffolds, suggesting that it is possible to lure metastasizing cells into a trap by imitating the bone marrow microenvironment. A similar strategy was adopted by Bersani et al. (2014). They utilized a polyacrylamide hydrogel coated with bone marrow stromal cells (BMSCs), which was able to capture metastasizing cells of prostate cancer.

De Vlieghere et al. (2015) took a slightly different approach to mimic a pre-metastatic niche by developing traps made of iron oxide-coated microparticles, encapsulating metabolically active cancer-associated fibroblasts (CAFs) (**Figure 1** Scheme II-3-c). The CAFs continuously deposited ECM composed of type I collagen and tenascine C, among others, creating an adhesive environment for disseminated cancer cells. The microparticles were implanted into the peritoneal cavities of mice with induced ovarian cancer. Twenty-four hours after the implantation, the microparticles were magnetically removed, and the adhesion of cancer cells on the microparticles was assessed. The treatment led to a delay in peritoneal metastasis and prolonged the animal survival.

Another variation in the strategy for recruiting metastasizing cancer cells was presented by Azarin et al. (2015). They developed a microporous scaffold from poly(lactide-co-glycolic acid) (PLGA) scaffold for *in vivo* capture of metastasizing breast cancer cells through the induction of a local immune response (**Figure 1** Scheme II-3-d). Indeed, it has been shown that immune cells are implicated in tumor cell recruitment (Qian et al., 2009; Qian et al., 2011). Here, they have either recruited immune cells into the scaffold by grafting the chemokine CCL22, which is known to induce migration of immune cells but not tumor cells, or incorporated the Gr1^hi^CD11b^+^ immune cells directly into the PLGA scaffold. By doing this, they were able to reduce the number of breast cancer cells that metastasized to the lung by 88%. Similarly, Rao et al. (2016) designed a poly(ε-caprolactone) (PCL)-based device with a slower degradation rate than PLGA scaffolds and investigated the immune response induced at the implantation site, the ability of the device to recruit metastatic cells for detection prior to colonization of organs as well as its influence on the survival of mice with induced breast cancer. Pelaez et al. (2018) further developed the strategy to enable the elimination of the attracted metastatic cells by noninvasive focal hyperthermia. To do so, they coupled metal discs to PCL microparticles to allow heat generation through electromagnetic induction using an oscillating magnetic field. The heat generation could be modulated conveniently by changing the size of the disc or the type of metal.

It has been shown that exosomes, which are vesicles involved in the transfer of information between cells, play a role in homing CTCs in the pre-metastatic niche (Peinado et al., 2012). De La Fuente et al. (2015) harnessed the potential of this knowledge and designed a three-dimensional scaffold with embedded exosomes extracted from the ascitic fluid of ovarian cancer patients (**Figure 1** Scheme II-3-e). The scaffold, called M-Trap, was implanted in the inner wall of the peritoneum of mice with a xenograft of human ovarian cancer in the peritoneal cavity. They showed that the scaffold could serve as the preferred site of metastasis, while a peritoneal carcinometastasis was observed in the absence of the M-Trap. Moreover, an increase in the mean survival was observed in the presence of the M-Trap (from 117.5 to 198.8 days), which was further improved by the removal of the scaffold (mean of survival of 309.4 days). The safety and performance of the M-Trap is currently being evaluated in a clinical trial involving female patients with stage IIIC ovarian cancer (ClinicalTrials.gov Identifier NCT03085238).

#### Chemoattraction of Tumor Cells

Migrating cells can make directional choices when presented with different migration pathways. *In vitro*, it has been shown that neutrophil-like cells can navigate through a microfabricated maze by following a chemical gradient (Skoge et al., 2016). Chemokines and their receptors are particularly involved in this navigation process. They are indeed responsible for the chemoattraction of various cells and could therefore be used to attract migrating tumor cells into a trap. Several receptors/chemokines have been identified to facilitate cancer cell migration. The most investigated one is SDF-1, also called CXCL12, which binds with high affinity to the CXCR4 and CXCR7 receptors. This chemokine is a pro-inflammatory mediator and is known to play a role in the recruitment of T cells, monocytes, and lympho-hemopoietic progenitor cells (Crump et al., 1997). Its overexpression has been linked to an increase in the invasiveness of ovarian cancer (Kajiyama et al., 2008), breast cancer (Bachelder et al., 2002; Zhan et al., 2016), and GBM (Barbero et al., 2003; Hira et al., 2017), among others [further details can be found elsewhere (Kryczek et al., 2007)]. In addition, despite being less well studied, CXCL16 and its receptor CXCR6 are also suspected to play a role in the migration of tumor cells. Wang et al. (2008) have shown that the expression of CXCR6 increases with the grade of prostate cancer. These results were supported by Lu et al. (2008) who observed that metastatic cells from prostate cancer overexpress the CXCR6 receptor. Moreover, CXCL16 have been shown to induce the migration and enhance the proliferation of CXCR6-expressing cancer cells *in vitro* (Darash-Yahana et al., 2009).

With this knowledge, several groups have tried to stop the migration of tumor cells by inhibiting chemokine receptors, particularly the CXCR4 receptor. A reduction in the migration of cancer cells has been observed *in vitro* (Brennecke et al., 2014; Chittasupho et al., 2017; Zheng et al., 2017). However, this has not been successfully replicated *in vivo*. Brennecke et al. found that the use of CXCR4 antibody 12G5 can reduce the number of osteosarcoma pulmonary metastases having a diameter of <0.1 mm but not those of larger dimensions (Brennecke et al., 2014). This finding can be explained by the fact that chemoattraction of cancer cells can be mediated by several pairs of chemokine–receptor interaction (for example, SDF-1 can bind either CXCR4 or CXCR7 or both, and CXCR6 can be activated by CXCL16). In addition, cells can activate the so-called compensation mechanisms *in vivo* to maintain their migration capacity. Indeed, it has been observed that neural progenitor cells (NPCs) are able to migrate in response to SDF-1 *via* the activation of the CXCR7 receptor in response to the blockade of the CXCR4 receptor (Chen et al., 2015). Therefore, in order to stop the migration of tumor cells completely, all receptors implicated in *in vivo* chemoattraction should be identified and blocked, making the task nearly impossible. Moreover, this strategy could only work if the tumor cells have yet to begin migrating. In the particular case of GBM, cancer cells usually have already invaded the surrounding tissues at the time of diagnosis (Giese et al., 2003). Thus, it may be more useful to direct the migration of cells toward a desired location instead of blocking the migration process altogether.

**Figure 4:**
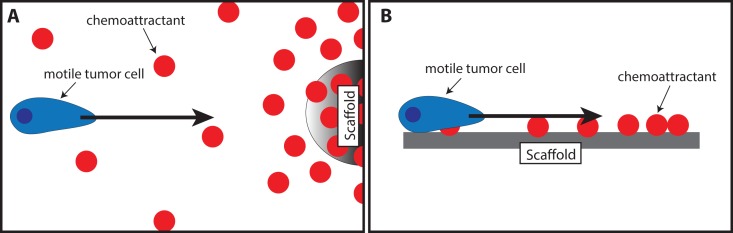
Illustration of the use of scaffolds to attract motile tumor cells by chemotaxis **(A)** or haptotaxis **(B)**.

Chemokines are already being used to attract cells into a scaffold for regenerative medicine purposes. The tumor trap concept can benefit from the existing knowledge in this field of application. Water-retaining polymer networks such as hydrogels and swellable matrices, which have been widely used in tissue engineering and regeneration, are pivotal platforms that are transferrable to the tumor trap application. For this purpose, biocompatible polymers capable of *in situ* formation of three-dimensional gels (Schesny et al., 2014; Shen et al., 2014; Addington et al., 2015) or matrices (Li et al., 2013; Yoon et al., 2013; Azarin et al., 2015; De Vlieghere et al., 2015; Ding et al., 2015; Autier et al., 2018) may be used to exert chemotaxis (based on a gradient of soluble attractant or repellant) or haptotaxis (based on a gradient of substrate-bound extracellular matrix proteins) (**Figure 4**). Of particular interest is the potential exploitation of the CXCR4-SDF-1 axis due to its prominent roles in regulating the migration of many types of cancer cells (Andreas et al., 2014; Schesny et al., 2014; Addington et al., 2015; Goffart et al., 2015). Examples of biomaterials used to deliver SDF-1 for regenerative medicine are presented in **Table 1**. Recently, the development of SDF-1-releasing scaffolds to attract tumor cells has received increasing attention. Giarra et al. (2018) designed a temperature-responsive gel loaded with SDF-1 based on methylcellulose (MC) or poloxamers with or without hyaluronic acid (HA) for the purpose of attracting CXCR4-expressing GBM cells (**Figure 1** Scheme II-3-f). Haji Mansor et al. (2018), on the other hand, encapsulated the chemokine in nanoparticles composed of PLGA and a (PEG)-PLGA copolymer to achieve sustained release (**Figure 1** Scheme II-3-g). However, in both papers, no *in vivo* assessment on the ability of SDF-1 to attract migrating cancer cells was performed.

**Table 1:** Strategies to load SDF-1 into different biomaterials.

Bonding strategy	Type of biomaterial	Composition	Target site for regeneration	Ref
**Adsorption**	Hydrogel	Hyaluronic acid	Cardiac tissue	(Purcell et al., 2012)
		PPCN	Wound healing	(Zhu et al., 2016)
	3D scaffold	Collagen	Cartilage	(Chen et al., 2015)
			Tendon	(Sun et al., 2018)
		Collagen/silk fibroin	Bone	(Hu et al., 2018)
		Hydroxyapatite	Bone	(Zhang et al., 2018)
		Decellularized skeletal muscle	Muscle	(Rajabi et al., 2018)
		Collagen/PLA	Bone	(Ritz et al., 2017)
		PLGA	Cartilage	(Wang et al., 2017)
	Membrane	PCL/gelatin	Bone	(Ji et al., 2013)
**Immobilization through specific heparin-mediated interaction**	Hydrogel	Heparin/PEG	Cardiac tissue	(Prokoph et al., 2012)
			Blood vessel	(Krieger et al., 2016)
			Cardiac tissue	(Baumann et al., 2013)
	3D scaffold	Heparin/PLCL	Blood vessel	(Shafiq et al., 2017)
		Heparin/PGS	Blood vessel	(Lee et al., 2013)
		Heparin/PLLA	Blood vessel	(Yu et al., 2012)
**Systems with nano/microparticles**	Microspheres	Alginate	Bone	(Xu et al., 2018)
	Hydrogel/Nanoparticles	Hydrogel: CS/GP nano: CS/CMCS	Bone	(Mi et al., 2017)
	Microcapsules	Dex-GMA/gelatin/PNIPAAm	Wound healing	(Chen et al., 2013)
	Particles	PLGA	Cardiac tissue	(Zamani et al., 2015)

PPCN, poly (polyethylene glycol citrate-co-N-isopropylacrylamide); PLA, polylactide; PLGA, poly(lactic-co-glycolic) acid; PCL, polycaprolactone; PEG, poly(ethylene-glycol); PLCL, poly(L-lactide-co-e-caprolactone) (PLCL); PGS, poly(glycerol sebacate); PLLA, poly(l-lactic acid); CS, chitosan; GP, beta-glycerol phosphate disodium salt; CMCS, carboxymethyl-chitosan; Dex-GMA, glycidyl methacrylated dextran; PNIPAAm, poly(N-isopropylacrylamide).

### Challenges Associated With the Clinical Translation of the Tumor Trapping Strategy

While promising preclinical results have been obtained from the use of tumor traps as a diagnostic and/or therapeutic tool, there are multiple issues that must be addressed before this approach can enter the clinic. Main concerns include identifying suitable means for *in vivo* monitoring of the recruitment of cancer cells into the scaffolds to allow one to decide on the optimal time point for killing the trapped cancer cells. Prolonged duration of cancer cell recruitment may lead to overcrowding of the tumor trap and subsequent cell escape, reducing the purpose of the synthetic niche to merely a “relay” for the cancer cells en route to their natural metastatic sites.

The incorporation of chemoattractant molecules such as SDF-1 into the tumor trap may also introduce additional complexities. In particular, there are concerns regarding the selectivity of SDF-1-mediated chemotaxis. Indeed, in addition to its role in recruiting cancer cells to local and distant sites of colonization, SDF-1 is also implicated in the homing of other cell lines such as immune cells and stem cells (Crump et al., 1997; Wang et al., 2017; Hu et al., 2018). Moreover, the potential off-target effects may also be exacerbated by the fact that this chemokine is known to be involved in various processes that support tumor progression, angiogenesis, metastasis, and survival (Teicher and Fricker, 2010). It is therefore necessary to study in more detail the effect of injecting such proteins near tumor cells *in vivo* and to carefully evaluate the entire risks before moving to the clinic.

Further down the development timeline, the most effective way to kill the recruited cancer cells should be elucidated. It may be tempting to suggest direct removal of the trap to achieve an immediate eradication of the disease. However, this approach will necessitate an additional surgery, a requirement that may be very difficult to fulfill especially in patients who are terminally ill. A less invasive solution would be to use stereotactic radiotherapy (SRT). SRT is a treatment where radiation beam is directed to a well-defined spot, usually the tumor site, from many different angles around the body. The procedure ensures the targeted site receives much higher dose of radiation than the surrounding tissues. At the moment, SRT seems to be a viable option for killing the trapped cancer cells. This said, other selective approaches should also be considered and evaluated.

## Conclusion

A good understanding of the escape pathways of a prey allows the hunter to capture it more efficiently; the same rule of thumb can be applied to tumor cells. Using this principle, it is possible to design tumor traps for diagnostic and/or therapeutic applications. For the latter purpose, it is necessary that the trapped cells are killed by the application of existing therapies. The different therapeutic strategies (surgery, chemotherapy, targeted therapy, …) may not be sufficient on their own to cure every cancer type, but they can be used in combination to achieve the best clinical outcomes. Jain et al. used a chemotherapeutic agent in the form of cyclopamine alongside their tumor trap to shrink down the size of GBM tumors (Jain et al., 2014). It would also be interesting to combine the trap with radiosensitizers, focus x-ray, or γ-ray microbeams. Since the trap would concentrate the tumor cells, the efficiency of chemo- and radiotherapies can potentially be improved, while the associated side effects are likely to decrease. Immunotherapy, which can be broadly described as the activation of immune cells to make them able to recognize and eliminate tumor cells, could also be used. Indeed, one of the major difficulties in immunotherapy is to make the cancer cells accessible to the activated immune cells. This is particularly true in the brain, as there is a need to overcome the blood–brain barrier (Lyon et al., 2017). If immune cells can be preloaded or attracted into the trap *via* chemoattraction, this will facilitate the killing of the trapped cancer cells. Indeed, immune cells are also sensitive to a gradient of chemokines such as SDF-1 (Krieger et al., 2016) and can therefore be recruited into the trap together with the cancer cells of interest. Overall, this bio-integrative approach can be seen as counterintuitive insofar as the factors governing the trapping of tumor cells are also involved in other signaling pathways that may lead to effects that are opposite to the initial will (Komarova, 2015). Our current knowledge on the mechanisms driving the migration of cancerous cells might not be sufficient to develop a trap that only impacts tumor cells in a safe manner. The translation to the clinic will therefore require further investigations on the efficacy and safety of such systems. Nevertheless, as Albert Einstein pointed out, “we do not solve problems with the modes of thought that have engendered them,” and this unique approach therefore deserves further investigations.

## Author Contributions

MN wrote the manuscript. CA-L and EG contributed to the conception and design of the work. MH, FB, CA-L and EG contributed to manuscript revision. All authors read and approved the submitted version.

## Funding

This work was supported by the “Institut National de la Santé et de la Recherche Médicale” (INSERM), the University of Angers (Angers, France), the MINECO (SAF2017-83118-R), the Agencia Estatal de Investigacion (AEI, Spain), and the Fondo Europeo de Desarollo Regional (FEDER). It is also related to the LabEx IRON “Innovative Radiopharmaceuticals in Oncology and Neurology” as part of the French government “Investissements d’Avenir” program, to the INCa (Institut National du Cancer) MARENGO consortium “MicroRNA agonist and antagonist Nanomedicines for GliOblastoma treatment: from molecular programmation to preclinical validation” through the PL-BIO 2014-2020 grant and to the MuMoFRaT project “Multi-scale Modeling & simulation of the response to hypo-Fractionated Radiotherapy or repeated molecular radiation Therapies” supported by “La Région Pays-de-la-Loire” and by the Cancéropôle Grand-Ouest (tumor targeting and radiotherapy network). MN was a Ph.D. student involved in the Erasmus Mundus Joint Doctorate program for Nanomedicine and pharmaceutical innovation (EMJD NanoFar) and received a fellowship from “La Région Pays-de-la-Loire.”

## Conflict of Interest Statement

The authors declare that the research was conducted in the absence of any commercial or financial relationships that could be construed as a potential conflict of interest.

## References

[B1] Addington C. P. Heffernan J. M. Millar-Haskell C. S. Tucker E. W. Sirianni R. W. Stabenfeldt S. E. (2015). Enhancing neural stem cell response to SDF-1alpha gradients through hyaluronic acid-laminin hydrogels. Biomaterials 72, 11–19. 10.1016/j.biomaterials.2015.08.041 26340314 PMC4593472

[B2] Aguado B. A. Bushnell G. G. Rao S. S. Jeruss J. S. Shea L. D. (2017). Engineering the pre-metastatic niche. Nat. Biomed. Eng. 1, 1–12. 10.1038/s41551-017-0077 PMC5628747 28989814

[B3] Andreas K. Sittinger M. Ringe J. (2014). Toward *in situ* tissue engineering: Chemokine-guided stem cell recruitment. Trends Biotechnol. 32, 483–492. 10.1016/j.tibtech.2014.06.008 25059433

[B4] Autier L. Clavreul A. Cacicedo M. L. Franconi F. Sindji L. Rousseau A. (2018). A new glioblastoma cell trap for implantation after surgical resection. Acta Biomater. 84, 268–279. 10.1016/j.actbio.2018.11.027 30465922

[B5] Azarin S. M. Yi J. Gower R. M. Aguado B. A. Sullivan M. E. Goodman A. G. (2015). *In vivo* capture and label-free detection of early metastatic cells. Nat. Commun. 6, 8094. 10.1038/ncomms9094 26348915 PMC4563812

[B6] Aznavoorian S. Stracke M. L. Krutzsch H. Schiffmann E. Liotta L. A. (1990). Signal transduction for chemotaxis and haptotaxis by matrix molecules in tumor cells. J. Cell Biol. 110, 1427–1438. 10.1083/jcb.110.4.1427 2324200 PMC2116083

[B7] Bachelder R. E. Wendt M. A. Mercurio A. M. (2002). Vascular endothelial growth factor promotes breast carcinoma invasion in an autocrine manner by regulating the chemokine receptor CXCR4. Cancer Res. 62, 7203–7206. 12499259

[B8] Barbero S. Bonavia R. Bajetto A. Porcile C. Pirani P. Ravetti J. L. (2003). Stromal cell-derived factor 1alpha stimulates human glioblastoma cell growth through the activation of both extracellular signal-regulated kinases 1/2 and Akt. Cancer Res. 63, 1969–1974. 12702590

[B9] Baumann P. Balasubramanian V. Onaca-Fischer O. Sienkiewicz A. Palivan C. G. (2013). Light-responsive polymer nanoreactors: a source of reactive oxygen species on demand. Nanoscale 5, 217–224. 10.1039/C2NR32380J 23154601

[B10] Bernstein J. J. Woodard C. A. (1995). Glioblastoma cells do not intravasate into blood vessels. Neurosurgery 36, 124–132. 10.1227/00006123-199501000-00016 7708148

[B11] Bersani F. Lee J. Yu M. Morris R. Desai R. Ramaswamy S. (2014). Bioengineered implantable scaffolds as a tool to study stromal-derived factors in metastatic cancer models. Cancer Res. 74, 7229–7238. 10.1158/0008-5472.CAN-14-1809 25339351 PMC4267901

[B12] Brennecke P. Arlt M. J. E. Campanile C. Husmann K. Gvozdenovic A. Apuzzo T. (2014). CXCR4 antibody treatment suppresses metastatic spread to the lung of intratibial human osteosarcoma xenografts in mice. Clin. Exp. Metastasis 31, 339–349. 10.1007/s10585-013-9632-3 24390633 PMC3915086

[B13] Brustugun O. T. Grønberg B. H. Fjellbirkeland L. Helbekkmo N. Aanerud M. Grimsrud T. K. (2018). Substantial nation-wide improvement in lung cancer relative survival in Norway from 2000 to 2016. Lung Cancer 122, 138–145. 10.1016/j.lungcan.2018.06.003 30032822

[B14] CELLSEARCH Circulating Tumor Cell (2019), https://www.cellsearchctc.com/ (accessed May 25, 2019).

[B15] Chen F. M. Lu H. Wu L. A. Gao L. N. An Y. Zhang J. (2013). Surface-engineering of glycidyl methacrylated dextran/gelatin microcapsules with thermo-responsive poly(N-isopropylacrylamide) gates for controlled delivery of stromal cell-derived factor-1&alpha. Biomaterials 34, 6515–6527. 10.1016/j.biomaterials.2013.05.014 23726519

[B16] Chen P. Tao J. Zhu S. Cai Y. Mao Q. Yu D. (2015). Radially oriented collagen scaffold with SDF-1 promotes osteochondral repair by facilitating cell homing. Biomaterials 39, 114–123. 10.1016/j.biomaterials.2014.10.049 25477178

[B17] Chen Q. Zhang M. Li Y. Xu D. Wang Y. Song A. (2015). CXCR7 mediates neural progenitor cells migration to CXCL12 independent of CXCR4. Stem Cells 33, 2574–2585. 10.1002/stem.2022 25833331 PMC4867224

[B18] Chen W. Allen S. G. Reka A. K. Qian W. Han S. Zhao J. (2016). Nanoroughened adhesion-based capture of circulating tumor cells with heterogeneous expression and metastatic characteristics. BMC Cancer 16, 614. 10.1186/s12885-016-2638-x 27501846 PMC4977622

[B19] Chiang S. P. H. Cabrera R. M. Segall J. E. (2016). Tumor cell intravasation. Am. J. Physiol. Cell Physiol. 311, C1–C14. 10.1152/ajpcell.00238.2015 27076614 PMC4967137

[B20] Chittasupho C. Anuchapreeda S. Sarisuta N. (2017). CXCR4 targeted dendrimer for anti-cancer drug delivery and breast cancer cell migration inhibition. Eur. J. Pharm. Biopharm. 119, 310–321. 10.1016/j.ejpb.2017.07.003 28694161

[B21] Crump M. P. Gong J. H. Loetscher P. Rajarathnam K. Amara A. Arenzana-Seisdedos F. (1997). Solution structure and basis for functional activity of stromal cell-derived factor-1; dissociation of CXCR4 activation from binding and inhibition of HIV-1. EMBO J. 16, 6996–7007. 10.1093/emboj/16.23.6996 9384579 PMC1170303

[B22] Darash-Yahana M. Gillespie J. W. Hewitt S. M. Chen Y. Y. K. Maeda S. Stein I. (2009). The chemokine CXCL16 and its receptor, CXCR6, as markers and promoters of inflammation-associated cancers. PLoS One 4, e6695. 10.1371/journal.pone.0006695 19690611 PMC2723911

[B23] De La Fuente A. Alonso-Alconada L. Costa C. Cueva J. Garcia-Caballero T. Lopez-Lopez R. (2015). M-Trap: exosome-based capture of tumor cells as a new technology in peritoneal metastasis. J. Natl. Cancer Inst. 107, 1–10. 10.1093/jnci/djv184 PMC4836824 26150590

[B24] De Vlieghere E. Gremonprez F. Verset L. Mariën L. Jones C. J. De Craene B. (2015). Tumor-environment biomimetics delay peritoneal metastasis formation by deceiving and redirecting disseminated cancer cells. Biomaterials 54, 148–157. 10.1016/j.biomaterials.2015.03.012 25907048

[B25] Ding Y. Wang Y. Opoku-Damoah Y. Wang C. Shen L. Yin L. (2015). Dual-functional bio-derived nanoparticulates for apoptotic antitumor therapy. Biomaterials 72, 90–103. 10.1016/j.biomaterials.2015.08.051 26344366

[B26] Dougan M. Dranoff G. Dougan S. K. (2018). Cancer immunotherapy: beyond checkpoint blockade. Annu. Rev. Cancer Biol. 3, 55–75. 10.1146/annurev-cancerbio-030518-055552 PMC10400018 37539076

[B27] Fabian D. Eibl M. d. P. G. P. E Alnahhas I. Sebastian N. Giglio P. Puduvalli V. (2019). Treatment of glioblastoma (GBM) with the addition of tumor-treating fields (TTF): a review. Cancers (Basel) 11, 1–12. 10.3390/cancers11020174 PMC6406491 30717372

[B28] FDA (2017a). FDA approval brings first gene therapy to the United States.

[B29] FDA (2017b). FDA approves CAR-T cell therapy to treat adults with certain types of large B-cell lymphoma.

[B30] Feins S. Kong W. Williams E. F. Milone M. C. Fraietta J. A. (2019). An introduction to chimeric antigen receptor (CAR) T-cell immunotherapy for human cancer. Am. J. Hematol. 94, S3–S9. 10.1002/ajh.25418 30680780

[B31] Fidler I. J. (1970). Metastasis: quantitative analysis of distribution and fate of tumour emboli labeled with 125I-5-iodo-2[prime]-deoxyuridine. J. Natl. Cancer Inst. 45, 773–782. 5513503

[B32] Giarra S. Ierano C. Biondi M. Napolitano M. Campani V. Pacelli R. (2018). Engineering of thermoresponsive gels as a fake metastatic niche. Carbohydr. Polym. 191, 112–118. 10.1016/j.carbpol.2018.03.016 29661298

[B33] Giese A. Bjerkvig R. Berens M. E. Westphal M. (2003). Cost of migration: invasion of malignant gliomas and implications for treatment. J. Clin. Oncol. 21, 1624–1636. 10.1200/JCO.2003.05.063 12697889

[B34] Giladi M. Schneiderman R. S. Voloshin T. Porat Y. Munster M. Blat R. (2015). Mitotic spindle disruption by alternating electric fields leads to improper chromosome segregation and mitotic catastrophe in cancer cells. Sci. Rep. 5, 1–16. 10.1038/srep18046 PMC4676010 26658786

[B35] Goffart N. Kroonen J. Di Valentin E. Dedobbeleer M. Denne A. Martinive P. (2015). Adult mouse subventricular zones stimulate glioblastoma stem cells specific invasion through CXCL12/CXCR4 signaling. Neuro Oncol. 17, 81–94. 10.1093/neuonc/nou144 25085362 PMC4483049

[B36] Gritsenko P. G. Ilina O. Friedl P. (2012). Interstitial guidance of cancer invasion. J. Pathol. 226, 185–199. 10.1002/path.3031 22006671

[B37] Haji Mansor M. Najberg M. Contini A. Alvarez-Lorenzo C. Garcion E. Jérôme C. (2018). Development of a non-toxic and non-denaturing formulation process for encapsulation of SDF-1α into PLGA/PEG-PLGA nanoparticles to achieve sustained release. Eur. J. Pharm. Biopharm. 125, 38–50. 10.1016/j.ejpb.2017.12.020 29325770

[B38] Hill A. McFarlane S. Johnston P. G. Waugh D. J. J. (2006). The emerging role of CD44 in regulating skeletal micrometastasis. Cancer Lett. 237, 1–9. 10.1016/j.canlet.2005.05.006 15979783

[B39] Hira V. V. V. Verbovšek U. Breznik B. Srdič M. Novinec M. Kakar H. (2017). Cathepsin K cleavage of SDF-1α inhibits its chemotactic activity towards glioblastoma stem-like cells. Biochim. Biophys. Acta - Mol. Cell Res. 1864, 594–603. 10.1016/j.bbamcr.2016.12.021 28040478

[B40] Hiratsuka S. Watanabe A. Aburatani H. Maru Y. (2006). Tumour-mediated upregulation of chemoattractants and recruitment of myeloid cells predetermines lung metastasis. Nat. Cell Biol. 8, 1369–1375. 10.1038/ncb1507 17128264

[B41] Hu Y. Ran J. Zheng Z. Jin Z. Chen X. Yin Z. (2018). Exogenous stromal derived factor-1 releasing silk scaffold combined with intra-articular injection of progenitor cells promotes bone-ligament-bone regeneration. Acta Biomater. 71, 168–183. 10.1016/j.actbio.2018.02.019 29524675

[B42] Hyun K.-A. Koo G.-B. Han H. Sohn J. Choi W. Kim S.-I. (2016). Epithelial-to-mesenchymal transition leads to loss of EpCAM and different physical properties in circulating tumor cells from metastatic breast cancer. Oncotarget 7, 24677–24687. 10.18632/oncotarget.8250 27013581 PMC5029733

[B43] Iacobucci G. (2019). Cancer survival in England: rates improve and variation falls. BMJ 365, l1532. 10.1136/bmj.l1532 30940615

[B44] Jain A. Betancur M. Patel G. D. Valmikinathan C. M. Mukhatyar V. J. Vakharia A. (2014). Guiding intracortical brain tumour cells to an extracortical cytotoxic hydrogel using aligned polymeric nanofibres. Nat. Mater. 13, 308–316. 10.1038/nmat3878 24531400

[B45] Ji W. Yang F. Ma J. Bouma M. J. Boerman O. C. Chen Z. (2013). Incorporation of stromal cell-derived factor-1α in PCL/gelatin electrospun membranes for guided bone regeneration. Biomaterials 34, 735–745. 10.1016/j.biomaterials.2012.10.016 23117215

[B46] Kajiyama H. Shibata K. Terauchi M. Ino K. Nawa A. Kikkawa F. (2008). Involvement of SDF-1alpha/CXCR4 axis in the enhanced peritoneal metastasis of epithelial ovarian carcinoma. Int. J. Cancer 122, 91–99. 10.1002/ijc.23083 17893878

[B47] Kaplan R. N. Riba R. D. Zacharoulis S. Bramley A. H. Vincent L. Costa C. (2005). VEGFR1-positive haematopoietic bone marrow progenitors initiate the pre-metastatic niche. Nature 438, 820–827. 10.1038/nature04186 16341007 PMC2945882

[B48] Kienast Y. Von Baumgarten L. Fuhrmann M. Klinkert W. E. F. Goldbrunner R. Herms J. (2010). Real-time imaging reveals the single steps of brain metastasis formation. Nat. Med. 16, 116–122. 10.1038/nm.2072 20023634

[B49] Kim E. G. Kim K. M. (2015). Strategies and advancement in antibody-drug conjugate optimization for targeted cancer therapeutics. Biomol. Ther. (Seoul) 23, 493–509. 10.4062/biomolther.2015.116 26535074 PMC4624065

[B50] Komarova N. L. (2015). Cancer: a moving target. Nature 525, 198–199. 10.2469/cfm.v20.n1.10 26308890

[B51] Krieger J. R. Ogle M. E. Faline-Figueroa J. Segar C. E. Temenoff J. S. Botchwey E. A. (2016). Spatially localized recruitment of anti-inflammatory monocytes by SDF-1α-releasing hydrogels enhances microvascular network remodeling. Biomaterials 77, 280-290. 10.1016/j.biomaterials.2015.10.045 PMC4698334 26613543

[B52] Kryczek I. Wei S. Keller E. Liu R. Zou W. (2007). Stroma-derived factor (SDF-1 / CXCL12) and human tumor pathogenesis. Am. J. Physiol. Cell Physiol. 292, 987–995. 10.1152/ajpcell.00406.2006 16943240

[B53] Kumar S. Weaver V. M. (2009). Mechanics, malignancy, and metastasis: the force journey of a tumor cell. Cancer Metastasis 28, 113–127. 10.1007/s10555-008-9173-4 PMC2658728 19153673

[B54] Lee J. T. Lee S. D. Lee J. Z. Chung M. K. Ha H. K. (2013). Expression analysis and clinical significance of CXCL16/CXCR6 in patients with bladder cancer. Oncol. Lett. 5, 229–235. 10.3892/ol.2012.976 23255926 PMC3525352

[B55] Lee K. W. Johnson N. R. Gao J. Wang Y. (2013). Human progenitor cell recruitment *via* SDF-1α coacervate-laden PGS vascular grafts. Biomaterials 34, 9877–9885. 10.1016/j.biomaterials.2013.08.082 24060423 PMC3882008

[B56] Levental K. R. Yu H. Kass L. Lakins J. N. Egeblad M. Erler J. T. (2009). Matrix crosslinking forces tumor progression by enhancing integrin signaling. Cell 139, 891–906. 10.1016/j.cell.2009.10.027 19931152 PMC2788004

[B57] Li W. Wang J. Ren J. Qu X. (2013). 3D graphene oxide-polymer hydrogel: near-infrared light-triggered active scaffold for reversible cell capture and on-demand release. Adv. Mater. 25, 6737–6743. 10.1002/adma.201302810 24123218

[B58] Li D. Cao Y. Li J. Xu J. Liu Q. Sun X. (2017). MiR-506 suppresses neuroblastoma metastasis by targeting ROCK1. Oncol. Lett. 13, 370–376. 10.3892/ol.2016.5442 28123576 PMC5245134

[B59] Li Z. Song W. Rubinstein M. Liu D. (2018). Recent updates in cancer immunotherapy: a comprehensive review and perspective of the 2018 China Cancer Immunotherapy Workshop in Beijing. J. Hematol. Oncol. 11, 1–15. 10.1186/s13045-018-0684-3 30577797 PMC6303854

[B60] Liu X. Chen D. Liu G. (2014). Overexpression of RhoA promotes the proliferation and migration of cervical cancer cells. Biosci. Biotechnol. Biochem. 78, 1895–1901. 10.1080/09168451.2014.943650 25104222

[B61] Lu Y. Wang J. Xu Y. Koch A. E. Cai Z. Chen X. (2008). CXCL16 functions as a novel chemotactic factor for prostate cancer cells in vitro. Mol. Cancer Res. 6, 546–554. 10.1158/1541-7786.MCR-07-0277 18344492

[B62] Lyon J. G. Mokarram N. Saxena T. Carroll S. L. Bellamkonda R. V. (2017). Engineering challenges for brain tumor immunotherapy. Adv. Drug Deliv. Rev. 114, 19–32. 10.1016/j.addr.2017.06.006 28625831 PMC5870902

[B63] MagForce, NanoTherm has the potential to tap into the US prostate cancer market as aunique focal treatment option, (2018).

[B64] Maier-Hauff K. Ulrich F. Nestler D. Niehoff H. Wust P. Thiesen B. (2011). Efficacy and safety of intratumoral thermotherapy using magnetic iron-oxide nanoparticles combined with external beam radiotherapy on patients with recurrent glioblastoma multiforme. J. Neurooncol. 103, 317–324. 10.1007/s11060-010-0389-0 20845061 PMC3097345

[B65] Mi L. Liu H. Gao Y. Miao H. Ruan J. (2017). Injectable nanoparticles/hydrogels composite as sustained release system with stromal cell-derived factor-1α for calvarial bone regeneration. Int. J. Biol. Macromol. 101, 341–347. 10.1016/j.ijbiomac.2017.03.098 28330754

[B66] Mishra A. Shiozawa Y. Pienta K. J. Taichman R. S. (2011). Homing of cancer cells to the bone. Cancer Microenviron. 4, 221–235. 10.1007/s12307-011-0083-6 21826451 PMC3234327

[B67] Nathanson S. D. (2003). Insights into the mechanisms of lymph node metastasis. Cancer 98, 413–423. 10.1002/cncr.11464 12872364

[B68] Paget S. (1889). The distribution of secondary growths in cancer of the breast. Lancet 133, 571–573. 10.1007/s12307-014-0163-5 2673568

[B69] Paszek M. J. Zahir N. Johnson K. R. Lakins J. N. Rozenberg G. I. Gefen A. (2005). Tensional homeostasis and the malignant phenotype. Cancer Cell 8, 241–254. 10.1016/j.ccr.2005.08.010 16169468

[B70] Paul C. D. Mistriotis P. Konstantopoulos K. (2017). Cancer cell motility: lessons from migration in confined spaces. Nat. Rev. Cancer 17, 131–140. 10.1038/nrc.2016.123 27909339 PMC5364498

[B71] Peinado H. Alečković M. Lavotshkin S. Matei I. Costa-Silva B. Moreno-Bueno G. (2012). Melanoma exosomes educate bone marrow progenitor cells toward a pro-metastatic phenotype through MET. Nat. Med. 18, 883–891. 10.1038/nm.2753 22635005 PMC3645291

[B72] Pelaez F. Manuchehrabadi N. Roy P. Natesan H. Wang Y. Racila E. (2018). Biomaterial scaffolds for non-invasive focal hyperthermia as a potential tool to ablate metastatic cancer cells. Biomaterials 166, 27–37. 10.1016/j.biomaterials.2018.02.048 29533788

[B73] Polyak K. Weinberg R. A. (2009). Transitions between epithelial and mesenchymal states: acquisition of malignant and stem cell traits. Nat. Rev. Cancer 9, 265–273. 10.1038/nrc2620 19262571

[B74] Prokoph S. Chavakis E. Levental K. R. Zieris A. Freudenberg U. Dimmeler S. (2012). Sustained delivery of SDF-1alpha from heparin-based hydrogels to attract circulating pro-angiogenic cells. Biomaterials 33, 4792–4800. 10.1016/j.biomaterials.2012.03.039 22483246

[B75] Provenzano P. P. Eliceiri K. W. Campbell J. M. Inman D. R. White J. G. Keely P. J. (2006). Collagen reorganization at the tumor-stromal interface facilitates local invasion. BMC Med. 4, 38. 10.1186/1741-7015-4-38 17190588 PMC1781458

[B76] Provenzano P. P. Inman D. R. Eliceiri K. W. Trier S. M. Keely P. J. (2008). Contact guidance mediated three-dimensional cell migration is regulated by Rho/ROCK-dependent matrix reorganization. Biophys. J. 95, 5374–5384. 10.1529/biophysj.108.133116 18775961 PMC2586586

[B77] Pulukuri S. Rao J. (2008). Martix matalloproteinase-1 promotes prostate tumor growth and metastasis. Int. J. Oncol. 32, 757–765. 10.3892/ijo.32.4.757 18360703 PMC2292413

[B78] Purcell B. P. Elser J. A. Mu A. Margulies K. B. Burdick J. A. (2012). Synergistic effects of SDF-1α chemokine and hyaluronic acid release from degradable hydrogels on directing bone marrow derived cell homing to the myocardium. Biomaterials 33, 7849–7857. 10.1016/j.biomaterials.2012.07.005 22835643 PMC3449064

[B79] Qian B. Deng Y. Im J. H. Muschel R. J. Zou Y. Li J. (2009). A distinct macrophage population mediates metastatic breast cancer cell extravasation, establishment and growth. PLoS One 4, e6562. 10.1371/journal.pone.0006562 19668347 PMC2721818

[B80] Qian B.-Z. Li J. Zhang H. Kitamura T. Zhang J. Campion L. R. (2011). CCL2 recruits inflammatory monocytes to facilitate breast-tumour metastasis. Nature 475, 222–225. 10.1038/nature10138 21654748 PMC3208506

[B81] Rajabi S. Jalili-Firoozinezhad S. Ashtiani M. K. Le Carrou G. Tajbakhsh S. Baharvand H. (2018). Effect of chemical immobilization of SDF-1α into muscle-derived scaffolds on angiogenesis and muscle progenitor recruitment. J. Tissue Eng. Regen. Med. 12, e438–e450. 10.1002/term.2479 28512922

[B82] Rankin E. B. Giaccia A. J. (2016). Hypoxic control of metastasis. Science 352, 175–180. 10.1126/science.aaf4405 27124451 PMC4898055

[B83] Rao S. S. Bushnell G. G. Azarin S. M. Spicer G. Aguado B. A. Stoehr J. R. (2016). Enhanced survival with implantable scaffolds that capture metastatic breast cancer cells *in vivo*. Cancer Res. 76, 5209–5218. 10.1158/0008-5472.CAN-15-2106 27635043 PMC5027988

[B84] Ritz U. Gerke R. Götz H. Stein S. Rommens P. M. (2017). A new bone substitute developed from 3D-prints of polylactide (PLA) loaded with collagen i: an in vitro study. Int. J. Mol. Sci. 18, 2569–2583. 10.3390/ijms18122569 PMC5751172 29186036

[B85] Sambi M. Bagheri L. Szewczuk M. R. (2019). Current challenges in cancer immunotherapy: multimodal approaches to improve efficacy and patient response rates. J. Oncol. 2019, 1–12. 10.1155/2019/4508794 PMC6420990 30941175

[B86] Schesny M. K. Monaghan M. Bindermann A. H. Freund D. Seifert M. Eble J. A. (2014). Preserved bioactivity and tunable release of a SDF1-GPVI bi-specific protein using photo-crosslinked PEGda hydrogels. Biomaterials 35, 7180–7187. 10.1016/j.biomaterials.2014.04.116 24875761

[B87] Seib F. P. Berry J. E. Shiozawa Y. Taichman R. S. Kaplan D. L. (2015). Tissue engineering a surrogate niche for metastatic cancer cells. Biomaterials 51, 313–319. 10.1016/j.biomaterials.2015.01.076 25771021 PMC4367489

[B88] Shafiq M. Kong D. Kim S. H. (2017). SDF-1α peptide tethered polyester facilitates tissue repair by endogenous cell mobilization and recruitment. J. Biomed. Mater. Res. A. 105, 2670–2684. 10.1002/jbm.a.36130 28571106

[B89] Shen Y. Abaci H. E. Krupsi Y. Weng L. Burdick J. A. Gerecht S. (2014). Hyaluronic acid hydrogel stiffness and oxygen tension affect cancer cell fate and endothelial sprouting. Biomater. Sci. 2, 655–665. 10.1039/c3bm60274e 24748963 PMC3987918

[B90] Shiozawa Y. Havens A. M. Jung Y. Ziegler A. M. Pedersen E. A. Wang J. (2008). Annexin II/annexin II receptor axis regulates adhesion, migration, homing, and growth of prostate cancer. J. Cell. Biochem. 105, 370–380. 10.1002/jcb.21835 18636554 PMC3614912

[B91] Shiozawa Y. Pedersen E. A. Taichman R. S. (2010). GAS6/Mer axis regulates the homing and survival of the E2A/PBX1-positive B-cell precursor acute lymphoblastic leukemia in the bone marrow niche. Exp. Hematol. 38, 132–140. 10.1016/j.exphem.2009.11.002 19922767 PMC2815170

[B92] Skoge M. Wong E. Hamza B. Bae A. Martel J. Kfataria R. (2016). A worldwide competition to compare the speed and chemotactic accuracy of neutrophil-like cells. PLoS One 11, 1–19. 10.1371/journal.pone.0154491 PMC4917115 27332963

[B93] Strebhardt K. Ullrich A. (2008). Paul Ehrlich’s magic bullet concept: 100 years of progress. Nat. Rev. Cancer 8, 473–480. 10.1038/nrc2394 18469827

[B94] Stupp R. Taillibert S. Kanner A. Read W. Steinberg D. M. Lhermitte B. (2017). Effect of tumor-treating fields plus maintenance temozolomide vs maintenance temozolomide alone on survival in patients with glioblastoma a randomized clinical trial. J. Am. Med. Assoc. 318, 2306–2316. 10.1001/jama.2017.18718 PMC5820703 29260225

[B95] Sun J. Mou C. Shi Q. Chen B. Hou X. Zhang W. (2018). Controlled release of collagen-binding SDF-1α from the collagen scaffold promoted tendon regeneration in a rat Achilles tendon defect model. Biomaterials 162, 22–33. 10.1016/j.biomaterials.2018.02.008 29428676

[B96] Taichman R. S. Cooper C. Keller E. T. Pienta K. J. Taichman N. S. Mccauley L. K. (2002). Use of the stromal cell-derived factor-1 / CXCR4 pathway in prostate cancer metastasis to bone. Cancer Res. 62 (6), 1832–1837. 11912162

[B97] Taphoorn M. J. B. Dirven L. Kanner A. A. Lavy-Shahaf G. Weinberg U. Taillibert S. (2018). Influence of treatment with tumor-treating fields on health-related quality of life of patients with newly diagnosed glioblastoma a secondary analysis of a randomized clinical trial. JAMA Oncol. 4, 495–504. 10.1001/jamaoncol.2017.5082 29392280 PMC5885193

[B98] Teicher B. A. Fricker S. P. (2010). CXCL12 (SDF-1)/CXCR4 pathway in cancer. Clin. Cancer Res. 16, 2927–2931. 10.1158/1078-0432.CCR-09-2329 20484021

[B99] Trama A. Bernasconi A. McCabe M. G. Guevara M. Gatta G. Botta L. (2019). Is the cancer survival improvement in European and American adolescent and young adults still lagging behind that in children? Pediatr. Blood Cancer 66, 1–9. 10.1002/pbc.27407 30124231

[B100] van der Sanden B. Appaix F. Berger F. Selek L. Issartel J.-P. Wion D. (2013). Translation of the ecological trap concept to glioma therapy: the cancer cell trap concept. Future Oncol. 9, 817–824. 10.2217/fon.13.30 23718302 PMC4788699

[B101] Wang J. Lu Y. Wang J. Koch A. E. Zhang J. Taichman R. S. (2008). CXCR6 induces prostate cancer progression by the AKT/mammalian target of rapamycin signaling pathway. Cancer Res. 68, 10367–10376. 10.1158/0008-5472.CAN-08-2780 19074906 PMC2884407

[B102] Wang C. Tong X. Yang F. (2014). Bioengineered 3D brain tumor model to elucidate the effects of matrix stiffness on glioblastoma cell behavior using PEG-based hydrogels. Mol. Pharm. 11, 2115–2125. 10.1021/mp5000828 24712441

[B103] Wang Y. Sun X. Lv J. Zeng L. Wei X. Wei L. (2017). Stromal cell-derived factor-1 accelerates cartilage defect repairing by recruiting bone marrow mesenchymal stem cells and promoting chondrogenic differentiation. Tissue Eng. Part A 23, 1160–1168. 10.1089/ten.tea.2017.0046 28478702 PMC6037190

[B104] Weiss L. (1992). Comments on hematogenous metastatic patterns in humans as revealed by autopsy. Clin. Exp. Metastasis 10, 191–199. 10.1007/BF00132751 1582089

[B105] Wirtz D. Konstantopoulos K. Searson P.C.P.P.C. (2011). The physics of cancer: the role of physical interactions and mechanical forces in metastasis. Nat. Rev. Cancer 11, 522. 10.1038/nrc3080 PMC3262453 21701513

[B106] Wolf K. Alexander S. Schacht V. Coussens L. M. von Andrian U. H. van Rheenen J. (2009). Collagen-based cell migration models in vitro and *in vivo*. Semin. Cell Dev. Biol. 20, 931–941. 10.1016/j.semcdb.2009.08.005 19682592 PMC4021709

[B107] Wolf K. Lindert M. Krause M. Alexander S. Riet J. Willis A. L. (2013). Physical limits of cell migration: control by ECM space and nuclear deformation and tuning by proteolysis and traction force. J. Cell Biol. 201, 1069–1084. 10.1083/jcb.201210152 23798731 PMC3691458

[B108] Wong S. Y. Hynes R. O. (2006). Lymphatic or hematogenous dissemination : perspective how does a metastatic tumor cell decide? Cell Cycle 5, 812–817. 10.4161/cc.5.8.2646 16627996 PMC1459485

[B109] Wozniak M. A. Desai R. Solski P. A. Der C. J. Keely P. J. (2003). ROCK-generated contractility regulates breast epithelial cell differentiation in response to the physical properties of a three-dimensional collagen matrix. J. Cell Biol. 163, 583–595. 10.1083/jcb.200305010 14610060 PMC2173660

[B110] Wu X. Y. Liu W. T. Wu Z. F. Chen C. Liu J. Y. Wu G. N. (2016). Identification of HRAS as cancer-promoting gene in gastric carcinoma cell aggressiveness. Am. J. Cancer Res. 6, 1935–1948. 10.1038/srep28044 27725900 PMC5043104

[B111] Wyckoff J. B. Jones J. G. Condeelis J. S. Segall J. E. (2000). A critical step in metastasis : *in vivo* analysis of intravasation at the primary tumor. Cancer Res. 60 (9), 2504–2511. 10811132

[B112] Wyckoff J. Wang W. Lin E. Y. Wang Y. Pixley F. Stanley E. R., (2004). A paracrine loop between tumor cells and macrophages is required for tumor cell migration in mammary tumors. Cancer Res. 64, 7022–7029. 10.1158/0008-5472.CAN-04-1449 15466195

[B113] Xu C. Xu J. Xiao L. Li Z. Xiao Y. Dargusch M. (2018). Double-layered microsphere based dual growth factor delivery system for guided bone regeneration. RSC Adv. 8, 16503–16512. 10.1039/C8RA02072H PMC9080232 35540506

[B114] Yoon H. J. Kim T. H. Zhang Z. Azizi E. Pham T. M. Paoletti C. (2013). Sensitive capture of circulating tumour cells by functionalized graphene oxide nanosheets. Nat. Nanotechnol. 8, 735–741. 10.1038/nnano.2013.194 24077027 PMC4017624

[B115] Yu J. Wang A. Tang Z. Henry J. Ping Lee B. Zhu Y. (2012). The effect of stromal cell-derived factor-1α/heparin coating of biodegradable vascular grafts on the recruitment of both endothelial and smooth muscle progenitor cells for accelerated regeneration. Biomaterials 33, 8062–8074. 10.1016/j.biomaterials.2012.07.042 22884813 PMC3488434

[B116] Zamani M. Prabhakaran M. P. Thian E. S. Ramakrishna S. (2015). Controlled delivery of stromal derived factor-1α from poly lactic-co-glycolic acid core-shell particles to recruit mesenchymal stem cells for cardiac regeneration. J. Colloid Interface Sci. 451, 144–152. 10.1016/j.jcis.2015.04.005 25897850

[B117] Zeng H. Chen W. Zheng R. Zhang S. Ji J. S. Zou X. (2018). Changing cancer survival in China during 2003–15: a pooled analysis of 17 population-based cancer registries. Lancet Glob. Health 6, e555–e567. 10.1016/S2214-109X(18)30127-X 29653628

[B118] Zhan Y. Zhang H. Liu R. Wang W. Qi J. Zhang Y. (2016). Eupolyphaga sinensis Walker ethanol extract suppresses cell growth and invasion in human breast cancer cells. Integr. Cancer Ther. 15, 102–112. 10.1177/1534735415598224 26242891 PMC5736081

[B119] Zhang H. Chen J. (2018). Current status and future directions of cancer immunotherapy. J. Cancer 9, 1773–1781. 10.7150/jca.24577 29805703 PMC5968765

[B120] Zhang B. Li H. He L. Han Z. Zhou T. Zhi W. (2018). Surface-decorated hydroxyapatite scaffold with on-demand delivery of dexamethasone and stromal cell derived factor-1 for enhanced osteogenesis. Mater. Sci. Eng. C Mater. Biol. Appl. 89, 355–370. 10.1016/j.msec.2018.04.008 29752108

[B121] Zheng N. Chen J. Li T. Liu W. Liu J. Chen H. (2017). Abortifacient metapristone (RU486 derivative) interrupts CXCL12/CXCR4 axis for ovarian metastatic chemoprevention. Mol. Carcinog. 56, 1896–1908. 10.1002/mc.22645 28277622

[B122] Zheng P. P. Kros J. M. Li J. (2018). Approved CAR T cell therapies: ice bucket challenges on glaring safety risks and long-term impacts. Drug Discov. Today. 23, 1175–1182. 10.1016/j.drudis.2018.02.012 29501911

[B123] Zhu C. Yago T. Lou J. Zarnitsyna V. I. McEver R. P. (2008). Mechanisms for flow-enhanced cell adhesion. Ann. Biomed. Eng. 36, 1–18. 10.1007/s10439-008-9464-5 18299992 PMC2633097

[B124] Zhu Y. Hoshi R. Chen S. Yi J. Duan C. Galiano R. D. (2016). Sustained release of stromal cell derived factor-1 from an antioxidant thermoresponsive hydrogel enhances dermal wound healing in diabetes. J. Control. Release 238, 114–122. 10.1016/j.jconrel.2016.07.043 27473766

